# Response of Circulating Free Cellular DNA to Repeated Exercise in Men with Type 1 Diabetes Mellitus

**DOI:** 10.3390/jcm13195859

**Published:** 2024-10-01

**Authors:** Konrad Walczak, Julia Grzybowska-Adamowicz, Robert Stawski, Olga Brzezińska, Agnieszka Zmysłowska, Dariusz Nowak

**Affiliations:** 1 Department of Internal Medicine and Nephrodiabetology, Medical University of Lodz, 90-549 Lodz, Poland; 2 Department of Clinical Genetics, Medical University of Lodz, 92-213 Lodz, Poland; 3 Department of Clinical Physiology, Medical University of Lodz, 92-215 Lodz, Poland; 4 Department of Rheumatology, Medical University of Lodz, 90-549 Lodz, Poland

**Keywords:** type 1 diabetes mellitus, exhaustive exercise, cell-free DNA, neutrophil extracellular traps, sport exercise

## Abstract

**Background:** Intense exercise leads to neutrophil extracellular traps (NETs) formation, which triggers cell disintegration. NET, as well as other processes of apoptosis, necrosis, and spontaneous secretion, result in increased levels of cell-free DNA (cf-DNA) in the circulation. An increment of cf-DNA is also observed in autoimmune diseases, such as type 1 diabetes mellitus (T1DM). Repeated exhaustive exercises are an impulse for physiological adaptation; therefore, in this case–control study, we compared the exercise-induced increase in cf-DNA in men with T1DM and healthy controls to determine the development of the tolerance to exercise. **Methods:** Volunteers performed a treadmill run to exhaustion at a speed matching 70% of their personal VO2 max at three consecutive visits, separated by a 72 h resting period. Blood was collected before and after exercise for the determination of plasma cell-free nuclear and mitochondrial DNA (cf n-DNA, cf mt-DNA) by real-time PCR, blood cell count and metabolic markers. **Results:** Each bout of exhaustive exercise induced a great elevation of cf n-DNA levels. An increase in cf mt-DNA was observed after each run. However, the significance of the increase was noted only after the second bout in T1DM participants (*p* < 0.02). Changes in cf-DNA concentration were transient and returned to baseline values during 72 h of resting. The exercise-induced increment in circulating cf n-DNA and cf mt-DNA was not significantly different between the studied groups (*p* > 0.05). **Conclusions:** Cf-DNA appears to be a sensitive marker of inflammation, with a lower post-exercise increase in individuals with T1DM than in healthy men.

## 1. Introduction

Type 1 diabetes mellitus (T1DM) is a chronic autoimmune condition with a lack of insulin and progressive hyperglycemia resulting from damage to pancreatic beta cells. Regular training is important for patients with T1DM. The positive effects could lower cardiovascular and overall mortality risks. The adaptation to repeated exercise involves a decrease in insulin resistance and an improvement in lipid levels and endothelial function. A sense of security is necessary for the patient’s commitment to regular activity. Modern technology enables the overcoming of the fear of hypoglycemia during training, and more and more people are able to exercise regularly and reap the positive health benefits [1]. Sportsmen with T1DM can compete at the highest level and even win medals. However, exhaustion might correspond with greater disadvantages for them than for healthy athletes. In order to achieve great results, training schedules and sports competitions require exhaustive exercises. Exhaustion is known to elevate pro-inflammatory cytokines in circulation, as well as increase the concentration of muscle damage markers [2,3].

Endurance and resistance exercises are known to influence metabolic changes and inhibit the progression of lifestyle-related diseases [4]. Repeated endurance exercises have been recognized to be an effective stimulus for physiological changes and remodeling in skeletal muscles [4,5]. Changes in intracellular signaling, transcriptional response, metabolic control, and mitochondrial density have been noted in this type of exercise. High-intensity training (repeated bouts of maximal exercise interspersed with short recovery intervals) is especially connected to elevated levels of glycolytic and oxidative enzymes, improvement in the development of capillaries in muscles, phosphocreatine resynthesis and regulation of K^+^, H^+^, and lactate levels. Nonetheless, strenuous exercise might lead to negative physiological effects such as an increase in inflammatory proteins and lactic acid accumulation [5,6]. Patients with diabetes are especially vulnerable to prolonged inflammation [7]. Intense exercise triggers neutrophil extracellular traps (NETs). The formation of NETs is an integral part of the body’s response to infection and tissue injury. They are involved in the pathogenesis of various autoimmune and cardiovascular diseases [8]. NETs are responsible for the disruption of cellular membranes. Due to the disintegration of the nuclear membrane, decondensed chromatin diffuses into the cytoplasm, leading to the elevation of circulating cell-free DNA (cf-DNA) in plasma [8,9,10]. Cf-DNA consists of two groups: cell-free nuclear DNA (cf n-DNA) from the nucleus and cell-free mitochondrial DNA (cf mt-DNA) from mitochondria located in the cytoplasm [11]. Tissue injury and inflammation, which are present in numerous diseases, may trigger elevation in cf n-DNA levels [2,12]. cf n-DNA may be an even better indicator of overtraining syndrome than C-reactive protein in patients with type 1 diabetes due to its sensitivity and proportional response to inflammation [13].

Increments of cf-DNA were observed in response to training in many disciplines [14,15,16,17]. In our recent study, we compared exercise-induced levels of cf-DNA in men with T1DM and healthy controls. However, the median exercise-induced increase in cf-DNA did not differ significantly in both groups [18]. Now, we would compare the response to repeated activity, which may give an insight into overreaching and decreased performance in athletes with T1DM and provide a marker for overtraining syndrome.

The purpose of this study was to evaluate exercise-induced changes in cf-DNA levels in young men with T1DM and matched healthy controls to determine the development of tolerance to exercise. We also compared markers of muscle damage and metabolic parameters induced by repeated exercise between those groups.

## 2. Material and Methods

### 2.1. Study Groups

The studied groups consisted of 14 patients with T1DM and 11 healthy men as controls. All volunteers were recreational sportsmen. Participants with T1DM were patients selected from the Diabetes (Central Clinical Hospital of the Medical University of Lodz, Poland). The control group was gathered from members of the Medical University of Lodz, which were selected from the database of the Academic Laboratory of Movement and Human Physical Performance “DynamoLab” at the same university. Participants had to meet the inclusion criteria: age between 25 and 45 years and provide written informed consent prior to the beginning of the study. The exclusion criteria were cigarette smoking, alcohol and illicit drug abuse, recent infectious or inflammatory diseases, and consumption of dietary supplements or any systemic medication within the last 3 months, similar to our previous study [3]. However, this time comparison of cf-DNA response between T1DM volunteers and matched healthy control was investigated. The volunteers with T1DM were not excluded due to complications of diabetes (not associated with chronic inflammatory responses) and their treatment, as well as arterial hypertension management with Ramipril (an angiotensin-converting enzyme inhibitor) at a daily dose of no more than 10 mg. All participants agreed to maintain dietary habits throughout the study and to adhere to the protocol instructions (RNN/95/14/KB). The results from three repeated runs completed by healthy controls have already been published. However, we present these results to compare them with results from the T1DM group. The first bout with both groups was also described [18]. Nonetheless, we consider adding two more runs beneficial in order to present the trend. 

### 2.2. Study Protocol

During the study, 4 visits were planned, taking place on the 1st, 7th, 10th, and 13th day of the protocol. On the first visit (day 1), participants underwent spirometry tests and a treadmill VO2 max test. Each visit started with a physical examination, blood pressure measurement, and electrocardiography. The three subsequent visits were separated by a 72 h resting period. On the 7th, 10th, and 13th day, the participants performed a treadmill run to exhaustion at a speed matching 70% of their VO2 max. Venous blood (15 mL) was collected into three different vacutainer tubes (Becton Dickinson, Franklin Lakes, NJ, USA) within 5 min before and after each bout of the exercise. The three types of tubes were (1) tubes with EDTA for cf-DNA measurement and blood cell count, (2) with sodium oxalate and potassium fluoride to access lactate levels, and (3) with gel and clot activator for blood chemistry. The volunteers otherwise did not engage in any strenuous exercise (13 days), as described earlier [3,10]. The study was carried out in compliance with the principles outlined in the Declaration of Helsinki. The protocol was approved by the Ethics Committee of the Medical University of Lodz (RNN/95/14/KB), and written informed consent was obtained from all participants.

### 2.3. Determination of VO2 Max

Measurement of VO2 max was performed as a continuous incremental maximal exercise test on a treadmill (Trackmaster CP 425) with the interface Ultima CardiO2 PFX (gas exchange analysis system), which was connected to 12-lead wireless Mortara ECG (Medical Graphics Corporation, St Paul, MN, USA), as described previously [3]. VO2 max was established for each participant when the three following criteria were met: (a) plateau in the O2 uptake despite increased speed performed by the runner, (b) respiratory exchange ratio exceeding 1.10, and (c) peak heart rate higher than 90% of age-predicted maximum (220–age) [19].

### 2.4. Execution of Treadmill Exhaustive Exercise

Between 7:00 and 8:00 a.m., all participants had their usual breakfast, and those with T1DM administered insulin doses determined by their individual carbohydrate intake and expected strenuous exercise. At 10:00 a.m. (after medical examination and short warm-up), participants performed running exercises on the treadmill (Trackmaster CP425) with a speed matching 70% of their VO2 max, and the incline of the treadmill was 1.5%. The participants ran until volitional exhaustion, which was determined when volunteers were unable to continue the run despite receiving encouragement from the testing staff. The Polar chest strap H7 was used to monitor heart rate. Mineral water was the only drink allowed during the run. Before and just after the strenuous run samples of venous blood were taken as well as blood pressure and body weight were measured. Each volunteer provided a separate written informed consent for each of the three exhaustive exercises.

### 2.5. Cell-Free DNA Extraction

cf-DNA was isolated from 400 μL plasma using a QIAamp DNA Blood Mini Kit (Qiagen GmbH, Hilden, Germany) with elution into 40 μL TE buffer according to manufacturer instructions, as previously described [3,10].

### 2.6. Real-Time PCR for the Measurement of cf n-DNA and cf mt-DNA in Plasma

Quantitative real-time PCR (qPCR) was performed for the quantification of cf n-DNA and cf mt-DNA isolated from plasma in the Central Scientific Laboratory “CoreLab” of the Medical University of Lodz. For cf n-DNA, the glyceraldehyde 3-phosphate-dehydrogenase (GAPDH) gene was used, and for the cf mt-DNA—mitochondrially encoded ATP synthase 8 (MT-ATP 8) gene. The method, execution of calibration curie as well as the sequences of the primers were described previously [3,20,21].

Simultaneous multiplex qPCR was performed in duplicate using the 7900 HT Real-time PCR System (Applied Biosystems, 850 Lincoln Centre Drive, Foster City, CA, USA), as described earlier. Individual results were obtained as a mean from two separate runs and expressed in ng/mL for cf n-DNA and as genome equivalents (GE)/mL plasma (1 GE = 6.6 pg DNA) for cf mt-DNA [3,20,21].

### 2.7. Other Parameters

Complete blood count, lactic acid, glucose, lipid panel (total cholesterol—TC, high-density lipoprotein cholesterol—HDL-C, low-density lipoprotein cholesterol—LDL-C, triglycerides—TG), urea, creatinine, serum activities of creatine kinase (CK), sodium, potassium, chloride concentrations aspartate aminotransferase (AST), alanine aminotransferase (ALT), and concentrations of C-reactive protein (CRP) were determined routinely, similarly as described previously [3].

### 2.8. Statistical Analysis 

For statistical analysis the Statistica 13.1 (StatSoft) software was used. Results were expressed as a mean ± standard deviation (SD). Analysis of variance (ANOVA) for repeated observations or Friedman’s ANOVA was applied for the evaluation of variations in variables over time (before and after three subsequent running exercises) depending on data distribution, which was tested with Shapiro–Wilk’s W test. When ANOVA was statistically significant, the post hoc analyses were applied. Scheffe’s test or post hoc analysis for Friedman’s ANOVA (multiple comparisons at 2 different time points) was performed. For the comparison of continuous variables between the study group and the control, the Mann–Whitney U test was performed; the data did not meet the criteria for parametric tests. A *p*-value < 0.05 was regarded as significant.

## 3. Results

### 3.1. Characteristics of the Studied Groups and Exhaustive Treadmill Bouts

The studied groups consisted of 14 volunteers with T1DM and 11 healthy controls. Their demographic and clinical characteristics are presented in Table S1. The groups did not differ significantly in body mass, height, body mass index, and VO2 max. The healthy control group has a higher mean age of 5 years (*p* = 0.018) than those with T1DM subjects. However, taking the age range between 25 and 45 years, that difference did not bring clinical significance. All studied participants, except one, successfully completed the study protocol of three exhaustive exercises performed every three days. One patient with T1DM had to stop exercising, probably due to hypoglycemia. No other complications resulted in the early termination of treadmill running. In both groups, an increase in mean distance and duration of exercise with every following run was observed, leading to a significant difference between the first and third bout in T1DM volunteers (*p* = 0.016). All subjects presented significant increments of post-exercise heart rate and arterial pressure (Table 1). The mean decrease in body mass was about 0.7–0.8 kg in both groups due to dehydration (Table 1). In accordance with water loss, hematocrit hemoglobin levels showed a tendency to increase in response to the exhaustive run (Table 2). Taking that into consideration, all results from plasma or serum analysis were adjusted for hematocrit changes due to water loss during exercise. Both groups presented similar elevations in the number of erythrocytes. The increase in white blood cell (WBC) count and the number of granulocytes, monocytes, and lymphocytes were similar in both groups after a strenuous run (Table S2). The mean increment in the platelet number was significantly higher in the T1DM group than in the controls (Table S2). Mean platelet volume (MPV) was increased similarly to the healthy control; however, the volume itself was smaller in T1DM subjects. Red cell distribution width (RDW) presented the same pattern in the second and third bouts (Table S2).

### 3.2. Changes of Selected Markers of Muscle Damage and Metabolic Response to Three Repeated Bouts of Exhaustive Treadmill Exercise

Significant elevation in circulating CK, lactic acid, AST, ALT, creatinine, and urea were noted after exercise in both groups. Additionally, T1DM participants presented significant increments in sodium, potassium, and chloride ions, most likely due to dehydration. In the T1DM group, mean glucose levels measured after exercise tended to decrease, whereas in healthy individuals, glucose concentration increased (Table 1).

### 3.3. Changes in Plasma Concentrations of Cell-Free Nuclear and Mitochondrial DNA in Response to Exhaustive Treadmill Exercise

Before the first run mean level of cf n-DNA in a plasma was 4.87 ± 3.0 ng/mL in T1DM participants and 3.37 ± 1.52 ng/mL in controls. The mean concentration of cf n-DNA increased greatly after each bout of exhaustive exercise (Table 3). The concentration differs significantly between groups before the third bout and after the last two bouts (Table 4). cf mt-DNA concentration before the exercises was similar in both studied groups (Table 4). After each run, an increase in the mean concentration of cf mt-DNA was observed (Table 3), but the significance of the increment was only stated after the second bout in T1DM participants (*p* = 0.01963). Moreover, only in that bout did the concentration of cf mt-DNA between groups show a significant difference (Table 4). The increments of cf n-DNA and cf mt-DNA were transient and the values returned to pre-exercise levels after each 72-h rest period in all volunteers (Table 3). Furthermore, exercise-induced increment in circulating cf n-DNA and cf mt-DNA was not significantly different between the studied groups (Table 4). The comparison of cf n-DNA and cf mt-DNA between single bouts was statistically insignificant according to the ANOVA Friedmann test.

### 3.4. Correlations of Exercise-Induced Increase in cf n-DNA (Δcf n-DNA) and cf mt-DNA (Δcf mt-DNA) with Selected Parameters

Table 5 presents positive correlations between exercise-induced cf n-DNA increment and selected indicators of muscle damage and metabolic response to exercise. Significant positive correlations were also observed between exercise-induced increase in cf mt-DNA levels and selected variables (*p* < 0.05) (Table 6).

## 4. Discussion

Elevation in the concentration of cf-DNA was measured after each one of the repeated bouts of exhaustive exercise in both T1DM volunteers and healthy controls. In our previous work [18], we described comparable responses to exercise in cf n-DNA levels in T1DM subjects and healthy controls. In this study, we wanted to investigate the ability to develop tolerance in studied groups and the difference in this process, basing our suspicions on chronic inflammation of the pancreatic islets in T1DM [22]. Surprisingly, no significant difference in elevations in cf-DNA levels was noted between groups. Patients with T1DM were not significantly different from healthy controls, so such exercises, even repeatedly, appear to be safe for them. The shorter distance run by participants with T1DM may correspond to the lack of a significant difference in cf-DNA levels. Furthermore, the exclusion criteria excluded T1DM patients with inflammatory complications and ongoing infection. Both processes participate in the activation of NET formation. Moreover, T1DM volunteers were diagnosed many years ago. The mean duration of diabetes in this group was 15.7 years. The autoimmune processes from the onset of the disease were no longer active. Once again, the formation of NETs was not stimulated. Thus, the lack of participants with an active inflammatory response might have led to no noted difference in cf DNA levels.

Thereafter, the concentrations of cf n-DNA were similar before and after exercise in each bout, suggesting no development of tolerance to repeated bouts. The increase of cf n-DNA and cf mt-DNA presented the same tendency in both groups. The tremendous increase in cf n-DNA concentration after exhaustive exercise corresponds with findings in articles studying other disciplines [14,18,23,24,25]. As was established earlier, cf n-DNA showed that it had a high correlation with acute and strenuous exercise [26].

In addition, the level of cf n-DNA was more sensitive towards exhaustion and showed greater increments than cf mt-DNA. Such a great difference in elevation between both groups of cf-DNA might be caused by various factors: Firstly, a decrease in cf n-DNA integrity in response to exercise [10]; secondly, the peak of cf n-DNA elevation occurs within the first minutes after maximal work-load, while the cf mt-DNA rises more during rest. A significant increase in the latter one was observed 30 and 90 min post-exercise [27]. Δcf n-DNA positively correlated with more parameters and showed stronger correlations in both groups than Δcf mt-DNA, similar to previous results [18]. Moreover, glucose levels decreased in T1DM participants and increased in healthy controls [18]. Various mechanisms are activated during exercise to prevent hypoglycemia. Inhibition of insulin release, increased secretion of glucagon, catecholamines, and cortisol leading to hepatic gluconeogenesis and glycogenolysis in muscles and liver plays a crucial role in the maintenance of glucose concentration in the circulation. These processes can even slightly elevate glucose levels in healthy individuals [1,28,29]. In T1DM patients, regulatory hormone response is unbalanced, and glucose production from the liver can often not keep up with glucose use by skeletal muscles, which may lead to decreased glucose levels [1,28]. Although only one participant in this group was suspected of having hypoglycemic episodes during the study protocol, special attention is required when coaching patients with T1DM to detect and prevent this acute complication. Moreover, all T1DM volunteers took a dose of insulin adjusted to the expected exhaustive exercise, which protected them from hyperglycemia and led to stimulation by its metabolic changes. 

In addition, the elevation of cf n-DNA was tremendously higher than the increment in CK, creatinine, AST, ALT, lactic acid, and urea levels in both studied groups. Cf n-DNA rose after every bout of exercise, while other markers were not so consistent. ALT was elevated after the first bouts in both groups, and AST levels increased in the first and second bouts in healthy control. The results correspond with healthy controls [3].

Furthermore, the number of platelets was significantly higher, and MPV was smaller in T1DM subjects after every run. RDW was smaller in the aforementioned groups; however, it was significant before and after the last two bouts. Physical exercise is described to induce the movement of neutrophils from the marginated pool of cells to the circulating pool [30,31]. As stated in our previous study [3], the differences between these two pools (including DNA release in response to exercise) and the distribution of neutrophils between them are not fully known. Moreover, the half-life of circulating neutrophils is no longer than a few days [30], so it is possible that important parts of blood neutrophils are replaced by new ones between the bouts of exercise, which causes a similar cf n-DNA response to three repeated runs. This hypothesis requires confirmation in further clinical studies. Strenuous exercise is reported to lead to transient neutrophilia, monocytosis, lymphocytosis, and elevation of platelets, MPV, and RDW [32,33]. A higher number of platelets was described in individuals with diabetes and their hypersensitivity to exogenous stimulus [34,35], which might be the reason for their greater elevation in T1DM participants. To our best knowledge, the significantly smaller RDW and MPV were not observed in adults with T1DM. It is possible that our findings are due to our small, studied group.

To summarize, muscle injury markers and metabolic changes stimulated by strenuous exercise are observed alongside the higher levels of cf-DNA in circulation. The not fully understood mechanism of cf-DNA release should be emphasized. We cannot exclude the possibility of different processes of elevation of cf-DNA in the T1DM group. An interesting insight might be provided by indicating the tissue of origin for circulating cf-DNA in different groups during exercise. The fragments with specific epigenetic signatures retain information that allows the identification of cell and tissue type [2,12]. Cf-DNA released into the circulation during exercise in patients with T1DM is thought to be from WBCs [36]. The strong correlation of Δcf n-DNA with Δ WBC also corresponds with this hypothesis.

The strength of the study is a highly controlled laboratory setting, ensuring the consistency and reliability of the measurements taken. This allowed for precise monitoring of circulating cf n-DNA and cf mt-DNA levels, along with other metabolic and muscle damage markers. The research design included the simultaneous assessment of both circulating cf n-DNA and other markers, providing a comprehensive view of the physiological responses to repeated exhaustive exercise in T1DM patients and healthy controls. The inclusion of recreationally trained men makes the findings relevant to the population of individuals with T1DM who are physically active, thereby enhancing the practical applicability of the results. The study adds valuable insights into the response of T1DM patients to intense physical activity, a group that is often underrepresented in exercise physiology research.

There are a few limitations of the study. The relatively low number of participants limits the generalizability of the findings. While the study was sufficient to demonstrate significant changes in cf n-DNA and cf mt-DNA levels, the small sample size restricted the ability to explore more detailed correlations between variables. Moreover, previously conducted studies presented a similar number of participants [14,15,25,37,38]. Additionally, the small sample size led to variability in the data, such as differences in age, BMI, and levels of physical activity among participants. These discrepancies further limit the ability to draw broad conclusions and may affect the robustness of the findings. The study included only male volunteers, which may limit the applicability of the results to female athletes with T1DM. The initial intention to include both genders was hampered by the difficulty in recruiting sport-active women who met the inclusion criteria. The issue of gender related bias was described by Nuzzo [39]. The analysis was conducted at only two time points (before and just after the exhaustive treadmill runs), potentially missing important information about the kinetics of cf-DNA and other markers throughout the recovery period. Our study was focused on exploring the impact of short-term effects of exhaustive exercise. Further research with a longer follow-up period would be needed to thoroughly investigate the long-term effect of exhaustive exercise. Some aspects of the study relied on participants’ self-reporting, such as dietary habits and exercise outside the study protocol, which could introduce potential biases or inaccuracies in the data. However, it is important to note that these self-reported factors did not form the basis of the study’s key conclusions. The demanding nature of the three exhaustive runs may have influenced participant recruitment and adherence, especially among individuals with T1DM, which could contribute to the overall smaller sample size and the homogeneity of the study population.

## 5. Conclusions 

In conclusion, three repeated bouts of exhaustive exercise separated by a 72 h rest period caused a great increment of cf n-DNA and a moderate increase in cf mt-DNA in both T1DM volunteers and healthy controls. No signs of tolerance development were observed. Cf-DNA is a very sensitive marker of inflammation. There are no signs of its accumulation. After exercise, its increase is lower in the T1DM group than in healthy, average-trained men. In order to establish correlations between changes in cf n-DNA and cf mt-DNA levels and other variables, further studies should be conducted with a larger number of participants and both sexes, with a particular focus on T1DM. Further insight into differences in elevation of cf-DNA between volunteers with T1DM and healthy control should be continued. Research on epigenetic changes in cf-DNA may also provide valuable information. Recognizing how different tissues are affected by exercise in various diseases will enable the development of personalized guidelines for training in specific disorders. 

## Figures and Tables

**Table 1:** Significance in changes of monitored parameters, selected circulating markers of muscle damage, and metabolic response to repeated bouts of exhaustive exercise in average-trained males with TIDM and healthy controls. *HDL*—high-density lipoprotein, *LDL*—low-density lipoprotein, *CK*—creatine kinase, *AST*—aspartate aminotransferase, *ALT*—alanine aminotransferase, *CRP*—*C-*reactive protein. * *p* < 0.05; ** *p* < 0.01; *** *p* < 0.0001 results before vs after presented bouts.

Marker	Bouts of Exhaustive Treadmill Exercise						
		Patients with Type 1 Diabetes			Healthy Controls		
		1st Bout	2nd Bout	3rd Bout	1st Bout	2nd Bout	3rd Bout
Systolic blood pressure [mmHg]	before after	128.79 ± 8.85 157.14 ± 17.18 ***	127.50 ± 13.55 157.86 ± 17.51 ***	125.00 ± 8.99 155.36 ± 15.50 ***	126.82 ± 6.43 172.27 ± 20.90 **	127.72 ± 7.54 169.54 ± 13.13 **	121.82 ± 9.02 166.18 ± 13.58 **
Diastolic blood pressure [mmHg]	before after	84.64 ± 6.64 83.21 ± 7.23	82.50 ± 5.80 84.29 ± 7.30	80.71 ± 4.75 80.71 ± 8.05	80.00 ± 5.00 83.00 ± 11.87	80.45 ± 6.50 79.09 ± 9.70	74.54 ± 5.68 79.09 ± 11.36
Heart rate [beats/min]	before after	77.21 ± 8.79 184.93 ± 11.82 ***	78.43 ± 11.30 183.93 ± 8.75 ***	76.43 ± 9.25 179.21 ± 9.07 ***	71.64 ± 11.85 184.00 ± 10.66 **	76.18 ± 12.67 183.45 ± 11.21 **	69.36 ± 10.64 175.64 ± 12.43 **
Body mass [kg]	before after	88.59 ± 13.46 87.76 ± 13.34 ***	88.49 ± 13.20 87.72 ± 13.20 ***	88.24 ± 13.16 87.36 ± 12.99 ***	87.41 ± 14.44 86.68 ± 14.40 **	87.14 ± 14.36 86.31 ± 14.41 **	87.67 ± 14.76 85.71 ± 15.27 **
Lactic acid [mmol/L]	before after	1.96 ± 0.71 8.25 ± 3.48 ***	1.70 ± 0.49 7.99 ± 3.83 ***	2.90 ± 4.92 7.74 ± 3.98 **	1.72 ± 0.80 8.95 ± 4.85 **	1.42 ± 0.70 8.83 ± 5.29 **	1.56 ± 0.49 7.97 ± 4.71 **
Sodium level [mmol/L]	before after	135.73 ± 1.72 138.23 ± 2.79 **	136.29 ± 2.19 138.81 ± 2.64 ***	135.69 ± 3.14 138.17 ± 2.47 **	138.01 ± 1.46 139.97 ± 2.06	137.85 ± 1.31 140.08 ± 2.64	138.08 ± 1.60 139.75 ± 2.06
Potassium level [mmol/L]	before after	4.21 ± 0.25 4.42 ± 0.44	4.23 ± 0.29 4.55 ± 0.42 *	4.28 ± 0.31 4.57 ± 0.40 *	4.23 ± 0.27 4.70 ± 0.58	4.23 ± 0.22 4.61 ± 0.42	4.32 ± 0.23 4.55 ± 0.50
Chloride level [mmol/L]	before after	100.68 ± 1.82 101.74 ± 2.26	101.65 ± 1.47 102.95 ± 2.63 *	101.21 ± 1.88 102.60 ± 1.81 **	102.54 ± 1.44 102.38 ± 1.99	103.08 ± 1.31 103.24 ± 1.52	103.36 ± 1.91 103.05 ± 1.45
Glucose [mmol/L]	before after	11.69 ± 3.81 9.73 ± 3.87	10.64 ± 3.53 9.53 ± 3.28	11.0 ± 3.90 9.76 ± 3.38	5.20 ± 0.80 6.84 ± 1.88	5.94 ± 1.21 6.95 ± 1.95	5.12 ± 1.06 6.28 ± 1.87
Urea [mmol/L]	before after	5.91 ± 1.18 6.31 ± 1.13 ***	5.62 ± 1.23 5.97 ± 0.96 **	5.45 ± 1.25 5.80 ± 1.05 *	5.82 ± 1.24 6.42 ± 1.59 *	5.73 ± 1.15 6.36 ± 1.1.49 **	6.13 ± 1.75 6.69 ± 1.80
Creatinine [umol/L]	before after	89.92 ± 10.17 107.57 ± 13.19 ***	80.07 ± 21.82 105.93 ± 14.27 **	85.79 ± 6.61 104.36 ± 11.84 ***	84.82 ± 12.18 116.00 ± 7.16 **	84.27 ± 10.50 113.55 ± 11.64 **	85.82 ± 14.52 105.91 ± 13.23 **
Total cholesterol [mmol/L]	before after	4.80 ± 0.82 4.91 ± 0.76	4.74 ± 0.73 5.01 ± 0.73 **	4.96 ± 0.66 4.95 ± 0.68 **	5.64 ± 0.85 6.16 ± 0.99 **	5.41 ± 0.93 5.54 ± 1.44	5.35 ± 0.66 5.77 ± 0.63
HDL fraction [mmol/L]	before after	1.47 ± 0.3 1.50 ± 0.28	1.45 ± 0.24 1.49 ± 0.28	1.41 ± 0.24 1.49 ± 0.26 **	1.34 ± 0.17 1.45 ± 0.18 **	1.29 ± 0.17 1.39 ± 0.19 **	1.32 ± 0.13 1.40 ± 0.16 **
LDL fraction [mmol/L]	before after	2.69 ± 0.67 2.68 ± 0.59	2.75 ± 0.69 2.69 ± 0.58	2.69 ± 0.72 2.71 ± 0.56	3.42 ± 0.84 3.73 ± 0.90 **	3.47 ± 0.88 3.61 ± 0.77 **	3.26 ± 0.58 3.49 ± 0.53 **
triglycerides [mmol/L]	before after	1.36 ± 0.56 1.43 ± 0.52	1.29 ± 0.69 1.41 ± 0.69 *	1.45 ± 0.64 1.52 ± 0.63	1.99 ± 0.90 2.15 ± 1.04 **	1.43 ± 0.57 1.85 ± 0.78	1.71 ± 0.77 1.92 ± 0.74
AST [U/L]	before after	31.00 ± 12.93 34.36 ± 15.53 **	28.64 ± 12.53 33.50 ± 14.75 ***	28.71 ± 12.77 31.86 ± 15.64 **	26.34 ± 7.05 35.13 ± 20.09 **	29.00 ± 8.65 35.69 ± 18.22 **	32.48 ± 10.90 54.35 ± 71.27
ALT [U/L]	before after	27.07 ± 11.69 28.93 ± 12.27 **	26.29 ± 11.07 28.93 ± 11.00 ***	27.86 ± 11.11 28.29 ± 11.96	27.64 ± 8.13 33.35 ± 14.77 **	29.10 ± 10.28 32.70 ± 13.11 **	32.22 ± 11.59 47.98 ± 54.70
CK [U/L]	before after	267.21 ± 174.75 325.21 ± 211.31 ***	239.64 ± 157.03 289.14 ± 174.00 **	226.36 ± 128.69 275.00 ± 153.32 **	162.18 ± 66.56 210.82 ± 117.67 **	266.64 ± 226.80 301.82 ± 193.44 **	300.82 ± 138.37 348.36 ± 136.10 **
CRP [mg/L]	before after	1.64 ± 1.35 1.69 ± 1.34	1.44 ± 0.88 1.54 ± 0.93 *	1.53 ± 1.09 1.54 ± 1.01	0.99 ± 0.63 1.53 ± 1.35 **	1.31 ± 1.15 1.29 ± 1.35	1.73 ± 1.85 1.92 ± 2.00

**Table 2:** Changes of blood cell count in response to repeated bouts of exercise in average-trained males with T1DM and healthy controls. *WBC*—white blood cells, *RBC*—red blood cells, HGB—hemoglobin, HCT—hematocrit, *PLT*—platelets, *LYM*—lymphocytes, *MON*—monocytes, *GRA*—granulocytes, *MCV*—mean corpuscular volume, *MCH*—mean corpuscular hemoglobin, *MCHC*—mean corpuscular hemoglobin concentration, *RDW*—red blood cell distribution width, *MPV*—mean platelet volume, *PDW*—platelet distribution width. Results are expressed as mean and standard deviation. * *p* < 0.05; ** *p* < 0.01; *** *p* < 0.0001 results before vs after presented bouts.

Marker		Bouts of Exhaustive Treadmill Exercise					
		Patients with Type 1 Diabetes			Healthy Controls		
		1st Bout	2nd Bout	3rd Bout	1st Bout	2nd Bout	3rd Bout
WBC [10^3^/µL]	before after	6.50 ± 1.33 9.29 ± 2.43 ***	5.95 ± 1.22 8.68 ± 2.49 ***	6.37 ± 2.60 9.45 ± 2.79 ***	5.86 ± 0.63 9.45 ± 1.94 ***	5.68 ± 0.66 9.99 ± 2.95 **	5.76 ± 0.66 10.72 ± 3.37 **
RBC [10^6^/µL]	before after	5.06 ± 0.23 5.19 ± 0.32 *	4.93 ± 0.30 5.11 ± 0.28 **	4.86 ± 0.35 5.09 ± 0.36 **	5.17 ± 0.41 5.32 ± 0.43 **	5.09 ± 0.48 5.28 ± 0.46 **	4.89 ± 0.50 5.16 ± 0.51 **
HGB [g/dL]	before after	15.36 ± 0.83 15.79 ± 1.02 *	14.96 ± 0.93 15.58 ± 0.85 **	14.72 ± 1.01 15.38 ± 0.97 **	15.64 ± 1.05 16.06 ± 1.19 **	15.30 ± 1.05 15.87 ± 1.05 **	14.76 ± 1.29 15.47 ± 1.20 **
HCT [%]	before after	45.61 ± 2.30 46.70 ± 2.82	44.40 ± 2.96 46.06 ± 2.26 **	43.73 ± 3.21 45.84 ± 3.21 **	46.75 ± 3.46 48.13 ± 3.46 **	45.95 ± 3.56 47.79 ± 3.58 **	44.06 ± 3.76 46.67 ± 3.91 **
PLT [10^3^/µL]	before after	236.23 ± 0.83 313.15 ± 61.69 ***	238.07 ± 43.01 305.69 ± 59.07 ***	233.69 ± 41.03 312.92 ± 51.10 ***	208.36 ± 33.11 258.36 ± 40.25 ***	199.82 ± 27.87 248.55 ± 32.09 ***	199.45 ± 35.80 247.09 ± 49.82 **
LYM [10^3^/µL]	before after	1.72 ± 0.44 3.12 ± 1.35 **	1.71 ± 0.43 3.11 ± 1.41 **	1.88 ± 0.92 3.10 ± 1.48 **	1.68 ± 0.33 3.07 ± 0.89 ***	1.64 ± 0.30 3.15 ± 0.79 ***	1.57 ± 0.30 3.05 ± 0.73 ***
MON [10^3^/µL]	before after	0.21 ± 0.11 0.35 ± 0.17 **	0.19 ± 0.06 0.33 ± 0.16 **	0.22 ± 0.13 0.33 ± 0.19 **	0.20 ± 0.04 0.36 ± 0.09 ***	0.19 ± 0.05 0.38 ± 0.10 ***	0.22 ± 0.04 0.40 ± 0.12 **
GRA [10^3^/µL]	before after	4.56 ± 1.25 5.83 ± 1.64 ***	4.04 ± 1.05 5.24 ± 1.49 ***	4.26 ± 1.70 6.02 ± 1.77 ***	3.98 ± 0.73 6.02 ± 1.91 **	3.85 ± 0.72 6.46 ± 3.04 *	3.97 ± 0.71 7.27 ± 3.59 **
MCV [fl]	before after	90.15 ± 3.58 90.08 ± 3.38	90.07 ± 3.68 90.15 ± 3.63	90.15 ± 3.51 90.23 ± 3.42	90.45 ± 3.45 90.45 ± 3.96	90.55 ± 3.75 90.55 ± 3.80	90.27 ± 3.58 90.73 ± 3.35
MCH [pg]	before after	30.40 ± 1.78 30.49 ± 1.81	30.36 ± 1.65 30.48 ± 1.58	30.31 ± 1.65 30.28 ± 1.68	30.28 ± 1.21 30.18 ± 1.40	30.16 ± 1.51 30.15 ± 1.39	30.25 ± 1.49 30.05 ± 1.44 *
MCHC [g/dL]	before after	33.70 ± 0.94 33.82 ± 1.04	33.70 ± 0.86 33.81 ± 0.72	33.66 ± 0.77 33.60 ± 0.83	33.49 ± 0.46 33.34 ± 0.42	33.32 ± 0.64 33.25 ± 0.56	33.51 ± 0.55 33.14 ± 0.51 **
RDW [%]	before after	13.77 ± 0.52 13.94 ± 0.59 *	13.59 ± 0.46 13.76 ± 0.48	13.70 ± 0.43 13.85 ± 0.31	14.06 ± 0.39 14.30 ± 0.56	14.11 ± 0.54 14.34 ± 0.50	14.10 ± 0.42 14.26 ± 0.37
MPV [fL]	before after	7.50 ± 0.66 7.71 ± 0.73 **	7.71 ± 0.73 7.75 ± 0.74	7.51 ± 0.76 7.77 ± 0.74 *	8.24 ± 0.87 8.43 ± 0.97	8.33 ± 0.89 8.57 ± 0.80 *	8.44 ± 0.99 8.69 ± 1.02 **
PDW [fL]	before after	16.41 ± 1.29 16.23 ± 1.15	16.09 ± 0.95 15.82 ± 1.14	15.96 ± 1.30 15.92 ± 1.23	16.24 ± 1.11 16.34 ± 1.02	16.67 ± 1.47 16.34 ± 1.16	16.19 ± 0.96 16.46 ± 1.06

**Table 3:** Circulating cell-free DNA response to repeated bouts of exhaustive exercise in average-trained males with T1DM and healthy controls. *cf n-DNA*—cell-free nuclear DNA. *cf mt-DNA*—cell-free mitochondrial DNA. Results are expressed as mean and standard deviation.

Marker	Bouts of Exhaustive Treadmill Exercise					
	1st Bout		2nd Bout		3rd Bout	
	Before	After	Before	After	Before	After
cf n-DNA [ng/mL] in T1DM patients	4.87 ± 3.00	34.45 ± 33.45	4.37 ± 2.84	31.44 ± 25.92	4.43 ± 2.46	36.90 ± 49.00
cf n-DNA [ng/mL] in controls	3.37 ± 1.52	38.47 ± 28.89	4.12 ± 3.47	48.47 ± 27.47	3.1 ± 1.66	53.8 ± 41.89
cf mt-DNA [10^3^ GE/mL] in T1DM patients	403.70 ± 426.33	418.82 ± 413.54	255.89 ± 177.86	374.13 ± 436.16	378.78 ± 422.30	462.80 ± 455.23
cf mt-DNA [10^3^ GE/mL] in controls	354.62 ± 230.63	460.98 ± 343.11	228.85 ± 226.6	450.61 ± 240.07	176.28 ± 126.13	462.17 ± 329.36

**Table 4:** Comparison of concentration of circulating cell-free DNA before and after repeated bouts of exercise and exercise-induced increment in circulating cell-free DNA in each of repeated bouts of exercise in average-trained males with T1DM and healthy controls. *cf n-DNA* cell-free nuclear DNA. *cf mt-DNA* cell-free mitochondrial DNA. Calculated using the Mann–Whitney U test.

Marker	Statistical Significance of Markers’ *p*-Value					
	1st Bout		2nd Bout		3rd Bout	
	Before	After	Before	After	Before	After
**cf n-DNA [ng/mL]** **T1DMs vs controls**	0.112	0.511	0.504	0.093	0.071	0.087
**cf mt-DNA [10^3^ GE/mL] T1DM vs controls**	0.763	0.805	0.602	0.082	0.271	0.643
	**1st bout**		**2nd bout**		**3rd bout**	
**Δ** **cf n-DNA [ng/mL]** **T1DM vs controls**	0.338		0.056		0.093	
**Δ** **cf mt-DNA [10^3^ GE/mL]** **T1DM vs controls**	0.494		0.118		0.602	

**Table 5:** Correlations (r) between exercise-induced increments in cf n-DNA and selected variables in average-trained males with TIDM and healthy controls. *CK*—creatine kinase. *AST*—aspartate aminotransferase. *ALT*—alanine aminotransferase. *CRP*—*C-*reactive protein. *RBC*—red blood cells. *Hct*—hematocrit. *PLT*—platelets. *LYM*—lymphocytes. *MON*—monocytes. *GRA*—granulocytes. *MCV*—mean corpuscular volume. *MCH*—mean corpuscular hemoglobin. *MCHC*—mean corpuscular hemoglobin concentration. *RDW*—red blood cell distribution width. *MPV*—mean platelet volume. *PDW*—platelet distribution width *cf n-DNA*—cell-free nuclear DNA. Significant values are indicated in bold.

Variable	Exercise-Induced Increments in cf n-DNA		
	T1DM Volunteers (n = 42)	Healthy Controls (n = 33)	All Studied Subjects (T1DM Volunteers and Healthy Controls. n = 75)
Δ WBC	r = −0892 *p* = 0.589	**r = 0.6220** ***p* < 0.0001**	**r = 0.2385** ***p* = 0.0436**
Δ AST	r = −0.2601 *p* = 0.110	**r = 0.4713** ***p* = 0.0056**	**r = 0.2569** ***p* = 0.0282**
Δ CK	**r = 0.3386** ***p* = 0.0326**	**r = 0.5461** ***p* = 0.0010**	**r = 0.3494** ***p* = 0.0024**
Δ Creatinine	r = 0.2957 *p* = 0.068	**r = 0.4862** ***p* = 0.0041**	**r = 0.4291** ***p* = 0.0002**
Δ urine	**r = 0.3301** ***p* = 0.040**	**r = 0.3748** ***p* = 0.0316**	**r = 0.2490** ***p* = 0.0337**
Δ lost of body mass	r = 0.0991 *p* = 0.548	**r = 0.4394** ***p* = 0.0105**	**r = 0.2566** ***p* = 0.0284**
Δ lactic acid	**r = −0.3311** ***p* = 0.0369**	**r = 0.4325** ***p* = 0.012**	**r = 0.4469** ***p* = 0.001**
Body mass before run	**r = −0.5221** ***p* = 0.0005**	r = −0.374 *p* = 0.836	**r = −0.2539** ***p* = 0.0302**
Body mass after run	**r = −0.5312** ***p* = 0.0004**	r = −0.0647 *p* = 0.721	**r = −0.2848** ***p* = 0.0146**
Lactic acid (mmol/L) before run	r = −0.2042 *p* = 0.212	**r = −0.3516** ***p* = 0.0448**	**r = −0.3284** ***p* = 0.0046**
Sodium level (mmol/L) before run	r = 0.2506 *p* = 0.124	r = −0.1058 *p* = 0.558	**r = 0.2813** ***p* = 0.0159**
Glucose before run	r = 0.0866 *p* = 0.600	r = 0.1631 *p* = 0.365	**r = −0.2959** ***p* = 0.0110**
Creatinine before run	r = −0.1524 *p* = 0.354	**r = −0.5237** ***p* = 0.0018**	**r = −0.3108** ***p* = 0.0075**
Urine before run	**r = −0.3615** ***p* = 0.024**	r = 0.1064 *p* = 0.555	**r = −0.3241** ***p* = 0.0414**
ALT before run	**r = −0.4181** ***p* = 0.0073**	**r = 0.4102** ***p* = 0.0177**	**r = −0.3315** ***p* = 0.037**
Lactic acid (mmol/L) after run	**r = −0.3979** ***p* = 0.0110**	**r = −0.4699** ***p* = 0.006**	**r = −0.2628** ***p* = 0.0247**
Sodium level after run	**r = 0.5263** ***p* = 0.0005**	**r = 0.4107** ***p* = 0.0176**	**r = 0.4394** ***p* = 0.0001**
CK after run	r = −0.0099 *p* = 0.952	**r = 0.6050** ***p* = 0.001**	**r = 0.3146** ***p* = 0.0067**
Glucose after run	**r = −0.4890** ***p* = 0.0014**	r = −0.2092 *p* = 0.243	**r = −0.3440** ***p* = 0.003**
ALT after run	**r = −0.4689** ***p* = 0.0023**	**r = 0.4625** ***p* = 0.0067**	**r = 0.2215** ***p* = 0.062**
CRP after run	r = 0.0176 *p* = 0.915	**r = 0.4981** ***p* = 0.0032**	**r = 0.2437** ***p* = 0.039**
PLT before run	r = −0.2477 *p* = 0.128	r = 0.3348 *p* = 0.057	**r = 0.2566** ***p* = 0.0284**
LYM before run	r = −0.1826 *p* = 0.266	r = −0.1605 *p* = 0.372	**r = 0.2875** ***p* = 0.0136**
MON before run	r = −0.3311 *p* = 0.069	r = 0.2872 *p* = 0.105	r = −0.0235 *p* = 0.845
MCHC before run	**r = 0.5656** ***p* < 0.001**	r = −0.3029 *p* = 0.087	**r = 0.2490** ***p* = 0.0337**
RDW before run	r = −0.2863 *p* = 0.077	**r = −0.3695** ***p* = 0.0343**	**r = 0.2727** ***p* = 0.0196**
MPV before run	r = 0.2135 *p* = 0.192	**r = −0.4550** ***p* = 0.0078**	**r = 0.4291** ***p* = 0.0002**
PDW before run	r = 0.0615 *p* = 0.710	r = −0.3096 *p* = 0.080	**r = 0.4360** ***p* = 0.0001**
WBC after run	**r = 0.3386** ***p* = 0.0326**	**r = 0.6101** ***p* = 0.0002**	**r = 0.3494** ***p* = 0.0024**
RBC after run	**r = 0.3644** ***p* = 0.0208**	r = −0.0745 *p* = 0.680	**r = 0.3370** ***p* = 0.0036**
Hct after run	**r = 0.3255** ***p* = 0.0404**	r = −0.1555 *p* = 0.388	**r = 0.4506** ***p* < 0.0001**
PLT after run	**r = 0.6093** ***p* < 0.0001**	**r = 0.3844** ***p* = 0.0272**	r = −0.0321 *p* = 0.789
LYM after run	**r = 0.9916** ***p* < 0.0001**	r = −0.2450 *p* = 0.169	**r = 0.9905** ***p* < 0.0001**
MON after run	**r = 0.3717** ***p* = 0.0182**	r = 0.1499 *p* = 0.169	**r = 0.4511** ***p* < 0.0001**
GRA after run	**r = 1** ***p* < 0.0001**	**r = 0.6312** ***p* < 0.0001**	**r = 1** ***p* < 0.0001**
MCV after run	r = 0.2133 *p* = 0.192	r = −0.1475 *p* = 0.413	**r = 0.2569** ***p* = 0.0282**
MCH after run	**r = 0.3982** ***p* = 0.012**	r = −0.2463 *p* = 0.167	**r = 0.3080** ***p* = 0.0080**
MCHC after run	**r = 0.5021** ***p* = 0.001**	r = −0.2663 *p* = 0.360	**r = 0.3604** ***p* = 0.0017**

**Table 6:** Correlations (r) between exercise-induced increments in cf n-DNA and selected variables in average-trained males with TIDM and healthy controls. *cf mt—DNA* cell-free mitochondrial DNA. *RBC*—red blood cells. *HCT*—hematocrit. *MCH*—mean corpuscular hemoglobin. Significant values are indicated in bold.

Variable	Exercise-Induced Increments in mtDNA		
	T1DM Volunteers (n = 42)	Healthy Controls (n = 33)	All Studied Subjects (T1DM Volunteers and Healthy Controls. n = 75)
Diastolic blood pressure before run [mmHg]	r = 0.0050 *p* = 0.976	r = −0.3418 *p* = 0.052	**r = −0.2874** ***p* = 0.014**
Systolic blood pressure before run [mmHg]	r = −0.2315 *p* = 0.156	**r = −0.5302** ***p* = 0.002**	r = −0.1870 *p* = 0.113
Δ RBC	r = 0.1945 *p* = 0.235	**r = 0.3665** ***p* = 0.036**	**r = 0.2654** ***p* = 0.024**
Δ HCT	r = 0.1777 *p* = 0.279	**r = 0.3565** ***p* = 0.042**	**r = 0.2538** ***p* = 0.031**
Δ MCH	r = −0.2350 *p* = 0.150	r = −0.2507 *p* = 0.159	**r = −0.2523** ***p* = 0.032**
Δ Creatinine	**r = 0.3724** ***p* = 0.020**	r = 0.0585 *p* = 0.746	**r = 0.2762** ***p* = 0.019**
Δ lactic acid	**r = −0.4096** ***p* = 0.010**	r = 0.0219 *p* = 0.904	r = −0.2288 *p* = 0.052
Lactic acid (mmol/L) before run	**r = 0.3552** ***p* = 0.026**	r = −0.0818 *p* = 0.651	**r = 0.2509** ***p* = 0.034**
Creatinine before run	r = −0.3003 *p* = 0.063	r = −0.2286 *p* = 0.201	**r = −0.2740** ***p* = 0.020**
Urine before run	**r = −0.3681** ***p* = 0.021**	r = −0.1760 *p* = 0.327	**r = −0.2533** ***p* = 0.032**
Urine after run	**r = −0.3775** ***p* = 0.018**	r = −0.1180 *p* = 0.513	r = −0.2051 *p* = 0.082

## Data Availability

The data may be available to all interested persons, upon request to the corresponding author.

## Supplementary Materials

The following supporting information can be downloaded at https://www.mdpi.com/article/10.3390/jcm13195859/s1, Table S1: Characteristic of the studied groups of T1DM volunteers and healthy controls. *T1DM* type 1 diabetes mellitus. Results are expressed as mean and standard deviation. Table S2: Comparison of changes in blood cell count in response to repeated bouts of exercise in average-trained males with T1DM and healthy controls. Calculated using the Mann–Whitney U test. *WBC*—white blood cells, *RBC*—red blood cells, *Hgb*—hemoglobin, *Hct*—hematocrit, *PLT*—platelets, *LYM*—lymphocytes, *MON*—monocytes, *GRA*—granulocytes, *MCV*—mean corpuscular volume, *MCH*—mean corpuscular hemoglobin, *MCHC*—mean corpuscular hemoglobin concentration, *RDW*—red blood cell distribution width, *MPV*—mean platelet volume, *PDW*—platelet distribution width.

## References

[1] Codella R., Terruzzi I., Luzi L. (2017). Why should people with type 1 diabetes exercise regularly?. Acta Diabetol..

[2] Drag M.H., Kilpelainen T.O. (2021). Cell-free DNA and RNA-measurement and applications in clinical diagnostics with focus on metabolic disorders. Physiol. Genom..

[3] Stawski R., Walczak K., Kosielski P., Meissner P., Budlewski T., Padula G., Nowak D. (2017). Repeated bouts of exhaustive exercise increase circulating cell free nuclear and mitochondrial DNA without development of tolerance in healthy men. PLoS ONE.

[4] Egan B., Zierath J.R. (2013). Exercise metabolism and the molecular regulation of skeletal muscle adaptation. Cell Metab..

[5] Coffey V.G., Hawley J.A. (2007). The molecular bases of training adaptation. Sports Med..

[6] Bogdanis G.C. (2012). Effects of physical activity and inactivity on muscle fatigue. Front. Physiol..

[7] King G.L. (2008). The role of inflammatory cytokines in diabetes and its complications. J. Periodontol..

[8] Beiter T., Fragasso A., Hartl D., Niess A.M. (2015). Neutrophil extracellular traps: A walk on the wild side of exercise immunology. Sports Med..

[9] Sollberger G., Tilley D.O., Zychlinsky A. (2018). Neutrophil Extracellular Traps: The Biology of Chromatin Externalization. Dev. Cell.

[10] Stawski R., Walczak K., Perdas E., Wlodarczyk A., Sarniak A., Kosielski P., Meissner P., Budlewski T., Padula G., Nowak D. (2019). Decreased integrity of exercise-induced plasma cell free nuclear DNA—Negative association with the increased oxidants production by circulating phagocytes. Sci. Rep..

[11] Tepper C.G., Studzinski G.P. (1993). Resistance of mitochondrial DNA to degradation characterizes the apoptotic but not the necrotic mode of human leukemia cell death. J. Cell Biochem..

[12] Spector B.L., Harrell L., Sante D., Wyckoff G.J., Willig L. (2023). The methylome and cell-free DNA: Current applications in medicine and pediatric disease. Pediatr. Res..

[13] Fatouros I.G., Destouni A., Margonis K., Jamurtas A.Z., Vrettou C., Kouretas D., Mastorakos G., Mitrakou A., Taxildaris K., Kanavakis E. (2006). Cell-free plasma DNA as a novel marker of aseptic inflammation severity related to exercise overtraining. Clin. Chem..

[14] Atamaniuk J., Vidotto C., Kinzlbauer M., Bachl N., Tiran B., Tschan H. (2010). Cell-free plasma DNA and purine nucleotide degradation markers following weightlifting exercise. Eur. J. Appl. Physiol..

[15] Fatouros I.G., Jamurtas A.Z., Nikolaidis M.G., Destouni A., Michailidis Y., Vrettou C., Douroudos I.I., Avloniti A., Chatzinikolaou A., Taxildaris K. (2010). Time of sampling is crucial for measurement of cell-free plasma DNA following acute aseptic inflammation induced by exercise. Clin. Biochem..

[16] Haller N., Helmig S., Taenny P., Petry J., Schmidt S., Simon P. (2018). Circulating, cell-free DNA as a marker for exercise load in intermittent sports. PLoS ONE.

[17] Tug S., Mehdorn M., Helmig S., Breitbach S., Ehlert T., Simon P. (2017). Exploring the Potential of Cell-Free-DNA Measurements After an Exhaustive Cycle-Ergometer Test as a Marker for Performance-Related Parameters. Int. J. Sports Physiol. Perform..

[18] Walczak K., Stawski R., Perdas E., Brzezinska O., Kosielski P., Galczynski S., Budlewski T., Padula G., Nowak D. (2021). Circulating cell free DNA response to exhaustive exercise in average trained men with type I diabetes mellitus. Sci. Rep..

[19] Howley E.T., Bassett D.R., Welch H.G. (1995). Criteria for maximal oxygen uptake: Review and commentary. Med. Sci. Sports Exerc..

[20] Xia P., An H.X., Dang C.X., Radpour R., Kohler C., Fokas E., Engenhart-Cabillic R., Holzgreve W., Zhong X.Y. (2009). Decreased mitochondrial DNA content in blood samples of patients with stage I breast cancer. BMC Cancer.

[21] Kohler C., Radpour R., Barekati Z., Asadollahi R., Bitzer J., Wight E., Burki N., Diesch C., Holzgreve W., Zhong X.Y. (2009). Levels of plasma circulating cell free nuclear and mitochondrial DNA as potential biomarkers for breast tumors. Mol. Cancer.

[22] Clark M., Kroger C.J., Tisch R.M. (2017). Type 1 Diabetes: A Chronic Anti-Self-Inflammatory Response. Front. Immunol..

[23] Beiter T., Fragasso A., Hudemann J., Niess A.M., Simon P. (2011). Short-term treadmill running as a model for studying cell-free DNA kinetics in vivo. Clin. Chem..

[24] Breitbach S., Sterzing B., Magallanes C., Tug S., Simon P. (2014). Direct measurement of cell-free DNA from serially collected capillary plasma during incremental exercise. J. Appl. Physiol..

[25] Atamaniuk J., Vidotto C., Tschan H., Bachl N., Stuhlmeier K.M., Muller M.M. (2004). Increased concentrations of cell-free plasma DNA after exhaustive exercise. Clin. Chem..

[26] Vittori L.N., Tarozzi A., Latessa P.M. (2019). Circulating Cell-Free DNA in Physical Activities. Methods Mol. Biol..

[27] Ohlsson L., Hall A., Lindahl H., Danielsson R., Gustafsson A., Lavant E., Ljunggren L. (2020). Increased level of circulating cell-free mitochondrial DNA due to a single bout of strenuous physical exercise. Eur. J. Appl. Physiol..

[28] Cockcroft E.J., Narendran P., Andrews R.C. (2020). Exercise-induced hypoglycaemia in type 1 diabetes. Exp. Physiol..

[29] Evans P.L., McMillin S.L., Weyrauch L.A., Witczak C.A. (2019). Regulation of Skeletal Muscle Glucose Transport and Glucose Metabolism by Exercise Training. Nutrients.

[30] Tak T., Tesselaar K., Pillay J., Borghans J.A., Koenderman L. (2013). What’s your age again? Determination of human neutrophil half-lives revisited. J. Leukoc. Biol..

[31] Athens J.W., Haab O.P., Raab S.O., Mauer A.M., Ashenbrucker H., Cartwright G.E., Wintrobe M.M. (1961). Leukokinetic studies. IV. The total blood, circulating and marginal granulocyte pools and the granulocyte turnover rate in normal subjects. J. Clin. Investig..

[32] Traiperm N., Gatterer H., Burtscher M. (2013). Plasma electrolyte and hematological changes after marathon running in adolescents. Med. Sci. Sports Exerc..

[33] Lippi G., Salvagno G.L., Danese E., Skafidas S., Tarperi C., Guidi G.C., Schena F. (2014). Mean platelet volume (MPV) predicts middle distance running performance. PLoS ONE.

[34] Sobczak A.I.S., Stewart A.J. (2019). Coagulatory Defects in Type-1 and Type-2 Diabetes. Int. J. Mol. Sci..

[35] Zaccardi F., Rocca B., Rizzi A., Ciminello A., Teofili L., Ghirlanda G., De Stefano V., Pitocco D. (2017). Platelet indices and glucose control in type 1 and type 2 diabetes mellitus: A case-control study. Nutr. Metab. Cardiovasc. Dis..

[36] Lui Y.Y., Chik K.W., Chiu R.W., Ho C.Y., Lam C.W., Lo Y.M. (2002). Predominant hematopoietic origin of cell-free DNA in plasma and serum after sex-mismatched bone marrow transplantation. Clin. Chem..

[37] Nasi M., Cristani A., Pinti M., Lamberti I., Gibellini L., De Biasi S., Guazzaloca A., Trenti T., Cossarizza A. (2016). Decreased Circulating mtDNA Levels in Professional Male Volleyball Players. Int. J. Sports Physiol. Perform..

[38] Velders M., Treff G., Machus K., Bosnyak E., Steinacker J., Schumann U. (2014). Exercise is a potent stimulus for enhancing circulating DNase activity. Clin. Biochem..

[39] Nuzzo J. (2021). Volunteer Bias and Female Participation in Exercise and Sports Science Research. Quest.

