# Supplementary material for: Response of Circulating Free Cellular DNA to Repeated Exercise in Men with Type 1 Diabetes Mellitus
Source: J Clin Med. 2024 Oct 1;13(19):5859. doi: 10.3390/jcm13195859 (PMC11477321; doi:10.3390/jcm13195859)
Supplement: Supplementary file 1 [file jcm-13-05859-s001.zip › jcm-3147441-supplementary.pdf]

Supplemental Table S1

**Demographic/clinical variables**	**Volunteers with T1DM**	**Healthy controls**	**p value**
Age [years]	29.28±5.38	34.8±5.49	0.018
Body mass [kg]	89.0±12.49	86.9±13.89	0.511
Height [cm]	178.43±7.88	182.45±7.57	0.310
Body mass index	24.90±3.03	23.76±3.16	0.311
VO2max [mL/kg/min]	46.7±5.62	49.63±4.82	0.366

Supplemental Table S1.Characteristic of the studied groups of T1DM volunteers and healthy controls. *T1DM* type 1 diabetes mellitus. Results are expressed as mean and standard deviation.

Supplemental Table S2

**Marker**	**Statistical significant of markers p value**					
**T1DM vs controls**	1st bout		2nd bout		3rd bout	
	before	after	before	after	before	after
**WBC [10^3/µl]**	0.228	1.000	1.000	0.207	0.955	0.361
**RBC [10^6/µl]**	0.494	0.331	0.277	0.424	0.733	0.494
**HGB [g/dl]**	0.392	0.569	0.277	0.392	0.361	0.569
**HCT [%]**	0.361	0.167	0.150	0.150	0.331	0.392
**PLT [10^3/µl]**	0.207	0.035	0.082	0.018	0.082	0.015

**LYM [10^3/µl]**	0.955	0.820	0.691	0.569	0.649	0.392
**MON [10^3/µl]**	0.955	0.649	1.000	0.228	0.608	0.134
**GRA [10^3/µl]**	0.207	0.910	0.910	0.459	0.955	0.569
**MCV [fl]**	0.776	0.865	0.776	0.820	0.955	0.776
**MCH [pg]**	1.000	0.608	0.865	0.733	0.955	0.865
**MCHC [g/dl]**	0.459	0.072	0.167	0.026	0.955	0.150
**RDW [%]**	0.134	0.167	0.015	0.013	0.030	0.013
**MPV [fl]**	0.041	0.047	0.106	0.035	0.026	0.035
**PDW [fl]**	0.910	0.955	0.186	0.252	0.608	0.277

Supplemental Table S2. Comparison of changes in blood cell count in response to repeated bouts of exercise in average-trained males with T1DM and healthy controls. Calculated using U Manna-Whitney test. WBC white blood cells, *RBC* red blood cells, *Hgb* hemoglobin, *Hct* hematocrit, *PLT* platelets, *LYM* lymphocytes, *MON* monocytes, *GRA* granulocytes, *MCV* mean corpuscular volume, *MCH* mean corpuscular hemoglobin, *MCHC* mean corpuscular hemoglobin concentration, *RDW* red blood cell distribution width, *MPV* mean platelet volume, *PDW* platelet distribution width.
